# Exploration of Entropy Pair Functional Theory

**DOI:** 10.3390/e24050603

**Published:** 2022-04-26

**Authors:** Clifton C. Sluss, Jace Pittman, Donald M. Nicholson, David J. Keffer

**Affiliations:** 1 Department of Materials Science & Engineering, University of Tennessee, Knoxville, TN 37996, USA; csluss@vols.utk.edu (C.C.S.); jpittm11@vols.utk.edu (J.P.); 2 Department of Physics and Astronomy, University of North Carolina, Asheville, NC 28803, USA

**Keywords:** entropy pair functional, iron, copper, silicon, modified embedded-atom method, Tersoff, molecular dynamics simulation

## Abstract

Evaluation of the entropy from molecular dynamics (MD) simulation remains an outstanding challenge. The standard approach requires thermodynamic integration across a series of simulations. Recent work Nicholson et al. demonstrated the ability to construct a functional that returns excess entropy, based on the pair correlation function (PCF); it was capable of providing, with acceptable accuracy, the absolute excess entropy of iron simulated with a pair potential in both fluid and crystalline states. In this work, the general applicability of the Entropy Pair Functional Theory (EPFT) approach is explored by applying it to three many-body interaction potentials. These potentials are state of the art for large scale models for the three materials in this study: Fe modelled with a modified embedded atom method (MEAM) potential, Cu modelled with an MEAM and Si modelled with a Tersoff potential. We demonstrate the robust nature of EPFT in determining excess entropy for diverse systems with many-body interactions. These are steps toward a universal Entropy Pair Functional, EPF, that can be applied with confidence to determine the entropy associated with sophisticated optimized potentials and first principles simulations of liquids, crystals, engineered structures, and defects.

## 1. Introduction

In material science, simulation is the third pillar of research, providing a complementary tool to experiment and theory. An attractive feature of simulation is that unambiguous access to all atomic coordinates is available. Density Functional Theory (DFT) simulation is the tool of choice for small systems and short time scales. Simulation with optimized classical potentials is the tool of choice for larger systems and longer time scales. Molecular dynamics (MD) has become a routine computational tool for investigating the structural, thermodynamic and transport properties of materials. MD simulations using optimized classical potentials for systems up to 10^5^–10^6^ atoms can be performed on modest compute clusters, while larger simulations are possible with access to supercomputing facilities. In terms of time scale, MD simulations from 1 to 10 ns are routine, while longer simulations are again possible given more extensive simulation resources. Routine simulations with DFT Hamiltonians are limited to 100 s of atoms for 100 s of ps.

As interest grows in materials with engineered disorder at the atomic scale, the ability to simulate systems with a sufficient number of atoms to capture the disorder further motivates interest in large scale MD simulations, where the use of first principles forces remains infeasible [1,2]. MD simulation of multicomponent materials with atomic-scale disorder, such as high entropy alloys (HEAs) or entropy stabilized oxides (ESOs) are limited by two issues. First, MD simulations require as input interaction potentials that describe how each type of atom interacts with each other type of atom. The robust and rapid determination of highly accurate interaction potentials for alloys or ceramics with arbitrary numbers of components is an area receiving great research interest.

The second challenge, the one on which this paper is focused, centers on the routine determination of entropy via MD simulation. The industry standard for MD simulation is the open-source simulation software, LAMMPS [3]. LAMMPS can generate instantaneous values of many thermodynamic properties, including temperature, pressure, density, internal energy and enthalpy. Properties based on thermodynamic partial derivatives of the above properties, such as heat capacity or isothermal compressibility, can be obtained accurately with just a couple of simulations, using a centered finite difference approach. Mechanical properties, such as the elastic tensor or the bulk modulus, are also readily extracted. Structural properties, such as the radial distribution function (RDF) emerge from the straightforward post-processing of the trajectory file generated from an MD simulation. Algorithms for the determination of transport properties, such as diffusivities, shear viscosities or thermal conductivities, from both equilibrium and non-equilibrium MD simulations, abound. The reader is directed to the “examples” directory that accompanies the LAMMPS source code, which contains demonstration scripts for obtaining all of the properties listed above. The property that most resists straightforward determination in MD simulation is the entropy, and by extension the Helmholtz and Gibbs free energies.

Certainly, it is possible to calculate relative entropy differences through thermodynamic integration. However, this approach requires that a series of simulations be performed across the integration path. Examples of entropy differences that can be evaluated in this way are the entropy change due to a change in temperature at constant volume

(1) $$\Delta S=\int_{T_{1}}^{T_{2}}\frac{C_{v}}{T}dT$$

or the entropy change due to a change in volume at constant temperature

(2) $$\Delta S=\int_{V_{1}}^{V_{2}}{\left(\frac{\partial p}{\partial T}\right)}_{V}dV$$

which follows from the Maxwell relation

(3) $${\left(\frac{\partial S}{\partial V}\right)}_{T}={\left(\frac{\partial p}{\partial T}\right)}_{V}$$

The drawback to thermodynamic integration is the computational expense required to perform the additional simulations. In some instances, there may also be a human-hour cost setting up addition configurations corresponding to each point along the integration path.

There remains interest in the determination of absolute entropy from a single simulation. To date, work has focused on determining the entropy as a functional of the PCF, which can be generated from a single simulation. The Gibbs formulation of Boltzmann entropy assumes the probability density of the atoms of a system in real space is known [4].

(4) $$S=-k_{B}\underset{i}{\sum }p_{i}ln\;p_{i}$$

For the MD practitioner, computation of entropy from Equation (4) depends upon a method to calculate a probability distribution, $p_{i}$, from atomic position data obtained from simulations. Beginning with the Kirkwood approximation for the calculation of a discrete probability distribution, others have developed a probability density based on the RDF and a resulting functional for the direct calculation of entropy [5,6,7]. Kirkwood (*K*) entropy, $S_{K}^{x}$, provides a formulation of the absolute excess (*x*) entropy in the fluid state as a functional of the RDF.

(5) $$S_{K}^{x}\left[g\right]=-1+\underset{R\to \infty }{\lim}\frac{1}{2}\{-1+\rho \int_{0}^{\infty }drg\left(r\right)\left(lng\left(r\right)-\left(g\left(r\right)-1\right))\right\}$$

where g(r) is the RDF. It is important to note that Equation (5) excludes all dependence on correlations higher in order than pair correlations. Kirkwood is one approximation to $p_{i}$; note that every approximation to the entropy can be related back to at least one approximation to $p_{i}$. For example, recent work by Haung and Widom [8] utilizes a Gaussian ansatz for $p_{i}$ that is applicable to crystals. It is Gaussian in the sense that it is the exponential of a form quadratic in atomic displacements. Since each factor involves just two sites, they obtain entropy as a functional of the PCF. Their $p_{i}$ is an approximate $p_{i}$ that produces a harmonic oscillator entropy. Thus, entropy calculated in quasi-harmonic approximation, based on first principles simulation of phonons [9], can be thought of as evaluating Equation (4) with an approximate $p_{i}$.

Contemporary approaches to the calculation of absolute excess entropy include improvements to Kirkwood entropy and the use of RDFs determined by first principles MD simulations [10,11,12] and machine learning techniques [13]. Recently, an approximation to the universal functional for the calculation of absolute excess entropy for pair potential Hamiltonians, from classical molecular dynamics simulations, has been developed [14]. This entropy pair functional theory (EPFT) has been demonstrated to provide reasonable agreement with excess entropy values produced by thermodynamic integration of MD simulation results for the Johnson iron (BCC) pair potential [15] across the entire temperature range, from crystalline solids at temperatures as low as 1 K up through the liquid state to a state approaching the perfect gas at 10^7^ K. As it currently exists, the EPFT approach specifies a temperature independent functional of the PCF that returns the excess entropy. This single functional is constructed from subsidiary functionals that highlight specific traits of the PCF. The simplest of these functionals identifies the PCF as corresponding to a crystal or fluid. If the PCF corresponds to a crystal, the widths of the peaks of the PCF at lattice separation vectors can be quantified by the variance of separation vectors within each peak. If the PCF corresponds to a fluid, the PCF is isotropic and is equal to its spherical average RDF. We depend on several subsidiary functionals of the RDF, for example, $S_{K}^{x}\left[g\right]$ and the coordination number. Evaluating the excess entropy requires various special integrals involving the PCF at each temperature of interest. However, unlike thermodynamic integration, the EPFT holds the promise that an accurate approximation can be found to the universal functional, which would make numerous simulations outside the points of interest unnecessary.

Formally, the entropy of many-body potentials depends not just on the PCF, but also on the many-body correlations. However, it was proved that the error introduced by using the EPF is second order in many-body interactions [14]. Modern simulations are often first principles, or use many-body interactions based on a mix of first principles results and measured properties. The extent to which an EPF effectively models the entropy of systems, governed by non-pairwise potentials, remains an open question. We apply EPFT to three many-body systems in order to explore the accuracy and universality of EPFT. EPFT must meet this challenge if it is to be adopted as an alternative to thermodynamic integration. The goal of this work is to investigate the universality of the EPFT approach with the explicit target of generating the absolute entropy from the pair correlation function (PCF) of a single simulation.

In this work we specifically explore the applicability of the EPFT to FCC copper and BCC iron systems simulated with the modified embedded atom method (MEAM) potential [16] and diamond cubic silicon system utilizing the Tersoff potential [17]. These systems and potentials were chosen out of a desire to take initial steps in demonstrating and expanding the universality of the EPFT. Copper and the MEAM potential provide the opportunity to test the EPFT with a new crystal structure (FCC), while iron provides a more direct comparison between MEAM and the Johnson potential of the original EPFT work. Silicon introduces a third crystal structure (diamond cubic) and the Tersoff potential tests the pair potential assumption of the underlying theory with the inclusion of large three--body angular terms.

## 2. Theory

Nicholson et al. have provided an extensive derivation of the EPFT approach and situated it within the historical framework of the Kirkwood superposition approximation for the fluid state and the harmonic oscillator approximation for the solid state [14]. For a full accounting, the interested reader is directed to that work. Here, we provide a summary of important points necessary to make this document self-sufficient.

The Entropy Pair Functional in [14] builds upon the Kirkwood entropy in the fluid state by introducing two new functionals that correct for two problem areas of the Kirkwood entropy [5,6,7,18,19]. First a corrective functional $\tilde{\phi }\left[g\right]$ is introduced to ensure that excess entropy approaches zero as the system approaches a perfect gas at high temperature. This high temperature limit is a subject that has previously been investigated with great interest [5]. The second functional γ[g] provides a correction as liquid approaches crystallization, where the Kirkwood entropy diverges. With the incorporation of these two corrections, the modified Kirkwood entropy in the fluid state becomes

(6) $${\tilde{S}}_{K}^{x}\left[g\right]=\frac{1}{2}\tilde{\phi }\left[g\right]-1+\underset{R\to \infty }{\lim}\frac{1}{2}\{-1+\frac{\rho }{\gamma \left[g\right]}\int_{0}^{\infty }drg\left(r\right)\left(lng\left(r\right)-\left(g\left(r\right)-1\right))\right\}$$

A further examination of the corrective functionals $\tilde{\phi }\left[g\right]$ and γ[g] will be presented in the methods section of this paper.

EPFT also extends the Kirkwood entropy into the crystalline phase down to arbitrarily low temperature. Several forms of the functional for the crystal have been proposed. In this work, we focus on three forms that utilize self and pair correlations only. The reference entropy of the crystalline state, $S_{r}^{x}\left[g\right]$, depends only on the variance of an atom around its lattice position, $1/2\lambda_{00}^{2}$,

(7) $$S_{r}^{x}\left[g\right]=-1+\frac{3}{2}+\frac{3}{2}ln\frac{\lambda_{00}^{2}}{{\bar{\ell }}^{2}}$$

where ℓ=1/ρ and $\bar{\ell }=\ell /\sqrt{\pi }$. $S_{x}^{r}$ is said to be the reference entropy, which is then refined by the addition of terms that arise from neighboring atoms. The second form for solid entropy implemented here is also based on Equation (7), but utilizes the variance of first nearest neighbor distances, 1/2$\lambda_{01}^{2}$, instead of the variance of atoms around their ideal lattice positions, $1/2\lambda_{00}^{2}$. Comparing Equation (9) to Equations (5) and (7) it can be seen that it represents a direct analog of the Kirkwood liquid excess entropy for solid excess entropy.

(8) $$S_{II\;h}^{x}\left[g\right]=-1+\frac{3}{2}+\frac{3}{2}ln\frac{\lambda_{01}^{2}}{{\bar{\ell }}^{2}}$$

The third form for solid entropy we calculate was presented by Nicholson et al. as a connection between the Kirkwood entropy and the harmonic solid technique of Morris and Ho [20].

(9) $${\tilde{S}}_{I\;h-\mathrm{TT}}^{x}=S_{x}^{r}\left[g\right]+\frac{3}{2}ln\left(1+\frac{1}{2}\left(\sqrt{1-4{\left|\epsilon_{1}\right|}^{2}}-1\right)\right)$$

where $S_{x}^{r}$ is defined in (3) and $\epsilon_{1}$ provides an off-diagonal coupling term derived from the truncated correlation matrix

(10) $$\epsilon_{1}=-\frac{\frac{\lambda_{01}^{2}\left[\bar{g}\right]}{4}-\frac{\lambda_{00}^{2}\left[\bar{g}\right]}{2}}{\frac{\lambda_{00}^{2}\left[\bar{g}\right]}{2}}$$

These three forms of solid entropy represent upper bounds. Due to this, in practice, the lower of the values produced should be considered the best estimate of entropy.

Finally, it must be noted that any calculation of excess entropy is impacted by the accounting of additional degrees of freedom. While our treatment here is limited to the degrees of freedom of the atoms in a system, additional sources of entropy may be of great interest to other workers. For example, electrons contribute to the entropy on their own in several ways and, through their impact, on the degrees of freedom that describe the nuclei. This is particularly true for iron; in addition to electron-hole entropy there is entropy resulting from the formation of local moments [21,22,23]. For work such as this, it is important to note that EPFT applies with only minor changes when the scope of simulations is expanded to include other degrees of freedom, e.g., those associated with site occupation, as in alloys, or spin degrees of freedom, as in magnetic materials. EPFT is expanded by indexing $g_{\alpha ,\beta }\left(r,r^{\prime }\right)$ where α and β refer to the atomic number and local atomic moment orientation at each nucleus.

## 3. Methods

### 3.1. Molecular Dynamics Simulations

A suite of classical MD simulations was performed for copper, iron and silicon. LAMMPS [3] was used to perform simulations of the three materials. The MEAM potential for Fe and for FCC Cu [16], and the Tersoff potential for Si [17], were taken from the literature. For a given material, the density remained constant at all temperatures. The densities are reported in Table 1. A summary of the simulation size is included in Table 1 below. As can be seen in the table, the length of the cubic simulation volume in any dimension was in the order of 10^2^ Å. This size was necessary to be able to calculate the RDF up to a maximum value of 50 Å.

For each material, a set of 40 simulations was performed for reduced temperatures in the range from 0.001 to 5000, where the temperatures were normalized by the melting temperature reported for each potential in the literature. (See Table 1) The Nose-Hoover thermostat was used to maintain the target temperature in the canonical ensemble (NVT). The size of the time step in each simulation was determined based on energy conservation in simulations in the microcanonical (NVE) ensemble, performed explicitly for this purpose. The time steps used ranged from ~13 fs at the lowest temperatures, nominally 1 K, to ~1.6 × 10^−2^ fs at the highest temperatures, nominally 10^6^ K. In general, the total duration of the simulations was determined in order to ensure convergence of thermodynamic values. Simulations were run for a duration to produce convergence of thermodynamic values and sufficient variability for configurational statistics calculations. All simulations were at least 4.57 ps in length.

The thermodynamic data generated was used to determine the potential energy of the systems for each temperature. For each simulation, atomic coordinates were recorded every 100 timesteps and this trajectory data was used to calculate the pair correlation functions, and the statistical values used as input for the entropy functional.

The system sizes were chosen to meet three criteria that we established. First, we wanted to demonstrate EPFT on systems sizes comparable to those typically used by MD simulators. Second, the system dimensions provide for RDF calculation out to 50 Å, which is commensurate with the range of correlations most often provided by experiment. Finally, by working with sufficiently large systems, complicating terms involving 1/N are avoided by operating at the thermodynamic limit. The simulations required an average of 20 h of wall time to complete when run on 2 nodes. This performance is very practical for the typical researcher compared to DFT-based approaches, which require tens of thousands of memory-laden (>2 TB/core) nodes to simulate systems of comparable size [24]. Note that the computer resources needed to evaluate the entropy with EPFT by post-processing of the trajectories of any simulation is negligible. For comparison, calculation of the entropy through direct calculation of phonon frequencies is computationally more intense for systems of this size.

### 3.2. Target Entropy Development

#### 3.2.1. Thermodynamic Integration

To validate the results from the EPFT, a target entropy was calculated across the whole temperature range of the simulations for each system to use as the standard. The standard entropy from thermodynamic integration and EPFT entropy are based on exactly the same Hamiltonian as rendered by LAMMPS. This is a better standard for comparison than are experimental entropies. The average potential energy was calculated for each temperature and an equation for U(T) was fit to the data. See Appendix A. The derivative $C_{V}=dU/dT$ and integral $\Delta S=\int_{T_{0}}^{T_{1}}\frac{C_{V}}{T}dT$ were determined analytically and curves for each were generated. This process was repeated for each material for both liquid and solid phases. Since the volumes of the simulations are constant across temperatures, the heat capacity is the constant-volume heat capacity. As the energy term employed is the potential energy, the heat capacity that is generated is the excess heat capacity, which does not include the kinetic contribution of the perfect gas. Similarly, the entropy arising from the excess heat capacity is strictly the excess entropy. Due to the discontinuity in the potential energy and entropy at the melt temperature, the liquid and solid target entropy curves must be developed separately and then reconnected via the calculation of the entropy of fusion.

#### 3.2.2. Entropy of Fusion

The Gibbs Phase Rule states that the number of Degrees of Freedom (*DOF*) required to fully define a thermodynamic state of system composed of *C* components and φ phases is given by

(11) DOF=C−φ+2

In the case of a single component system and a two-phase, e.g., solid-liquid, equilibrium, there is only one degree of freedom. Often, this *DOF* is chosen as the temperature, though that is a choice made out of practical considerations, rather than a theoretical requirement. All other thermodynamic properties, including the pressure, chemical potential and density of the phases are defined once the temperature has been specified. Notably, the densities of the solid and liquid phases at equilibrium are not the same.

As the target entropy developed for this work is along an isochor, the two phases present at the melt temperature are not in equilibrium. Thus, the discontinuity in entropy between the solid and liquid in this series of simulations does not correspond to the entropy of fusion of two phases at equilibrium. This motivated an approach to calculate the entropy difference between a liquid at a state defined by temperature and density (T,ρ), and a solid at the same temperature and density.

The entropy difference between a liquid at a thermodynamic state defined by arbitrary temperature, $T_{1}$, and arbitrary density, $\rho_{1}$. and a solid at the same temperature and density can be broken into three terms that describe a thermodynamic path. Since entropy is a state variable, this calculation is independent of path.

(12) $$\Delta S_{tot}=\Delta S_{L}+\Delta S_{L\to S}+\Delta S_{S}$$

The thermodynamic path we invoke is as follows. In step 1, a liquid at $\left(T_{1},\rho_{1}\right)$ undergoes an isothermal compression (or expansion) to liquid at $\left(T_{1},\rho_{2}\right)$, denoted $\Delta S_{L}$. In step 2, a liquid at $\left(T_{1},\rho_{2}\right)$ undergoes a phase change to solid at $\left(T_{1},\rho_{3}\right)$, with which it is in thermodynamic equilibrium, denoted $\Delta S_{L\to S}$. In step 3, a solid at $\left(T_{1},\rho_{3}\right)$ undergoes an isothermal expansion (or compression) to solid at $\left(T_{1},\rho_{1}\right)$, denoted $\Delta S_{S}$.

We choose $T_{1}$ to be a temperature where coexistence of the liquid and solid is possible. We choose $\rho_{2}$ to correspond to the dependent liquid phase density at the equilibrium state uniquely defined by $T_{1}$. We choose $\rho_{3}$ to correspond to the dependent solid phase density at the equilibrium state uniquely defined by $T_{1}$. This path provides the entropy difference between a liquid at a thermodynamic state defined by arbitrary temperature, $T_{1}$, and arbitrary density, $\rho_{1}$. and a solid at the same temperature and density. Practically speaking, we chose the temperature to correspond to the melting temperature at one atmosphere reported in the literature and reported in Table 1. In this case, the density of the coexisting liquid and solid were known and the entropy of fusion was reported in the literature [25,26]. The terms describing the entropy change due to a change in density were computed via thermodynamic integration using Equation (2). If the integral in Equation (2) is approximated with the trapezoidal rule, then, for the liquid and solid phases, Equation (12) becomes

(13) $$\Delta S_{L}=\frac{1}{2}\left(\frac{1}{\rho_{2}}-\frac{1}{\rho_{1}}\right)\left({\left(\frac{\partial p}{\partial T}\right)}_{\rho_{2}}^{L@T_{1}}+{\left(\frac{\partial p}{\partial T}\right)}_{\rho_{1}}^{L@T_{1}}\right)$$

(14) $$\Delta S_{S}=\frac{1}{2}\left(\frac{1}{\rho_{1}}-\frac{1}{\rho_{3}}\right)\left({\left(\frac{\partial p}{\partial T}\right)}_{\rho_{1}}^{S@T_{1}}+{\left(\frac{\partial p}{\partial T}\right)}_{\rho_{3}}^{S@T_{1}}\right)$$

The thermodynamic partial derivative ${\left(\frac{\partial p}{\partial T}\right)}_{V}$ evaluated under four conditions, as specified in (13) and (14) was evaluated using the centered finite difference formula

(15) $${\left(\frac{\partial p}{\partial T}\right)}_{\rho_{1}}\approx \frac{p\left(T+\delta T,\rho_{1}\right)-p\left(T-\delta T,\rho_{1}\right)}{2\delta T}$$

where δT is a temperature offset, chosen to be sufficiently large to provide a reliable gradient, given the noise present in the pressure calculation. Each derivative requires two simulations.

#### 3.2.3. Pair Correlation Functions

For the liquid entropy, the entropy functional takes, as input, radial distribution functions of the type defined below [27]. RDFs used as input to the entropy functional were calculated with bins of width 10^−3^ Å utilizing an in-house code.

(16) $$\int_{0}^{\infty }\rho g\left(r\right)4\pi r^{2}dr=N-1\;\approx N$$

**Figure 1:**
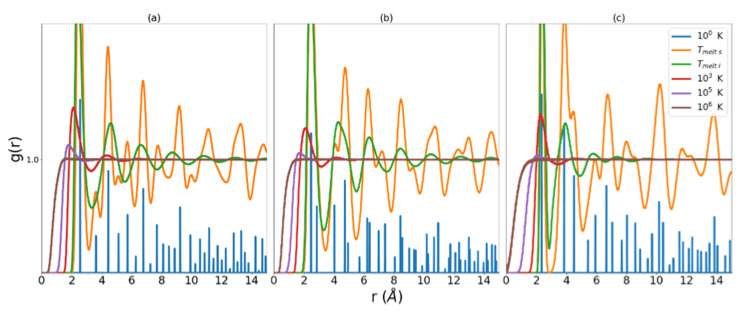
RDFs for MEAM copper (**a**), MEAM iron (**b**), and Tersoff silicon (**c**). Low temperature data has been scaled in order to highlight low r behavior at higher temperatures.

We return now to a more detailed discussion of the corrective functionals $\tilde{\phi }$ and γ introduced in the theory section. We consider first the error introduced in the approach to the perfect gas and begin with some observations about the RDF in general. It is evident from Equation (16) that the integral of the RDF is a volume that contains all the atoms under consideration less the volume of the central atom, from which the nearest neighbor distances are measured. This volume, referred to as the excluded volume, is shown to decrease as the temperature of the system increases. Examination of Figure 1 reveals that as temperature increases, the region in r that corresponds to zero probability of finding a nearest neighbor decreases. The RDF is defined such that at large r g(r) ≈1. This means that at sufficiently long distance there is unity probability of finding an atom in the next increment of volume.

#### 3.2.4. High Temperature Liquid Correction

We reintroduce a functional Q[g] that indicates the departure of the excluded volume from the origin; it is built upon the concept of the Wigner-Seitz cell. For the perfect crystal at 0 K each of the atomic cells in a system emerges as a Voronoi polyhedron (VP), centered on a single atom. The VP is defined to have faces that are perpendicular bisectors of the central atom and its neighbors. A corollary to this definition is that a point found inside the VP will have unity probability of being closer to the central atom than to any of the neighbors outside the VP. We adopt a probabilistic interpretation; at *T* = 0 (stationary atoms) *P*(*r*) = 1 for points inside the VP and 0 outside the VP; at finite temperature the VP changes over time but *P*(*r*) remains well defined.

The instantaneous VPs of the crystal evolves with time as atoms move at finite temperature. Eventually, the probability that a point displaced from atom *i* by *r* will be closer to atom *i* than to any other particle becomes spherical at melting. For this reason, a spherical probability was chosen as an approximate boundary for the measurement of the encroachment on the excluded volume as the temperature of the system approaches infinity. At infinite temperature the probability that a point a distance *r* from an atom is closer to that atom than any other atom is

(17) Pid(r)=limT→∞(1−r3Nrs3)(N−1)=exp(−(rRs)3)

where $R_{s}$ is the radius of a sphere of the atomic volume of the system, where the atomic volume is the inverse of the density. The derivation of this ideal gas probability is given in Appendix B. The intrusion, at any temperature, of neighboring atoms into the infinite temperature VP is given by

(18) $$I\left[g\right]=\int^{\;}4\pi r^{2}\rho P^{id}\left(r\right)g\left(r\right)dr$$

(19) $$Q\left[g\right]=\max\left(0,\frac{I\left[g\right]-I_{0}\left[g\right]}{1-I_{0}\left[g\right]}\right)$$

As T is lowered toward $T_{m}$ the peaks in g(r) become increasingly narrow. In the limit that the peaks have zero width, the intrusion of the nearest neighbors becomes

(20) $$I_{0}\left[g\right]=n\left[g\right]exp\left(-{\left(\frac{R_{p}\left[g\right]}{R_{s}}\right)}^{3}\right)$$

where $R_{p}$ is the radius of the first peak in $g\left(r\right)r^{2}$ and

(21) $$n\left[g\right]=2\int_{0}^{R_{p}}4\pi r^{2}\rho g\left(r\right)dr$$

$I_{0}\left[g\right]$ serves as a baseline for intrusion. Due to the fact that our results depended only weakly on $I_{0}\left[g\right]$, we made the simplifying, but not essential, choice that $I_{0}\left[g\right]$ is the minimum value of I[g], $I_{0}\left[g\right]=I_{min}$. This means that, in this work, the entropy calculation for the liquid is a single additional calculation of $I_{min}$ at the melting temperature. However, this calculation is less intensive than a full exploration of temperature required for thermodynamic integration. Consequently, the functional that characterizes the escape of the excluded volume from the origin is

(22) $$Q\left[g\right]=\frac{I\left[g\right]-I_{min}}{1-I_{min}}$$

Q[g] is used for the liquid phase only. It approaches zero at melting and 1−1/N in the perfect gas limit.

The high temperature correction $\tilde{\phi }\left[g\right]$ appearing in (6) is a functional of the functional Q[g].

(23) $$\tilde{\phi }\left[g\right]=Q+q_{1}Q\left(1-Q\right)+q_{2}Q^{2}\left(1-Q\right)$$

$\tilde{\phi }\left[g\right]$ possesses the same limits as Q[g], namely zero, at the melting temperature, and approaching one (within O(1/N)) at very high temperature. The parameters $q_{1}$ and $q_{2}$ fit the functional $\tilde{\phi }\left[g\right]$ϕ to the target entropy. These parameters, which are the same for all materials, allow the functional to match the behavior of the entropy at intermediate temperatures.

#### 3.2.5. Low Temperature Liquid Correction

**Figure 2:**
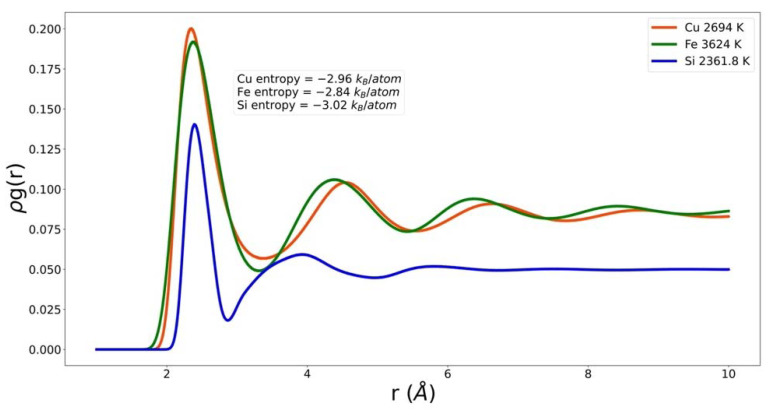
Neighbor density of copper, iron, and silicon at small r and similar excess entropy.

In Figure 2 one PCF for each system is shown multiplied by density, ρg(r). This quantity is referred to as the neighbor density. The three materials are very different. They correspond to very different temperatures. However, their common attribute is that they correspond to essentially the same excess entropy. One of the challenges for the EPF is to take these very different functions and return the same value. Note that by plotting the neighbor density, we have emphasized that the density of Si is significantly different from that of Cu and Fe, and that the coordination number of Si is much smaller than those of Cu and Fe. Furthermore, the nearest neighbor peak positions almost line up even though the atomic radius in Si is much smaller. The three systems have different packing fractions, $f_{p}$. The packing fraction of Si is considerably smaller than that of Fe and Cu.

Packing fraction, coordination number, intrusion, and $\lambda_{01}$ are descriptors of g(r) that depend only on the nearest neighbor peak; $\lambda_{00}$ can also be considered very local. On the other hand, the integrals of g(r)ln( g(r)) and ${\left(g\left(r\right)-1\right)}^{2}$ have contributions from all r; they emphasize the peaks, valleys, and their long-range persistence. This handful of physically motivated quantities provides a reasonable model of the entropy of the three systems studied here. Figure 2 shows that there are significant differences in the behavior in, for example, the first valley. These differences could be further exploited in the EPF. However, at this stage in the development of an EPF we prefer to show reasonable agreement with a small number of descriptors and parameters. These will naturally build up as we, and others, extend the range of universality by modeling additional systems.

As the liquid approaches $T_{m}$, the atoms in the system begin to be distributed near separations found in their ideal lattice. This can be seen in the ‘T melt l’ data series in Figure 1. Turning our attention now to the error in $S_{K}^{x}$ for the liquid in the region nearing crystallization, we observe, that as g(r) takes on the characteristics of a set of more and more discrete distributions around the ideal lattice separations, the natural log term in Equation (5) produces larger and larger negative values of excess entropy. This results in a gross under-estimation of excess entropy as the liquid cools toward crystallization. Any corrective functional must be constructed with this trend of g(r) in mind. In this case, Nicholson et al. proposed an indicator of the approach to crystalline structure and consequent correction measure constructed as follows

(24) h(r)=g(|r|)−1

(25) G=4πrh(r)

(26) $$\kappa \left[g\right]=\frac{\rho }{4\pi }\int^{\;}drG^{2}\left(r\right)$$

(27) $$\gamma \left[g\right]=1+q_{0}\kappa [g]$$

In liquid Fe near melting, a typical atom is surrounded by about 14 neighbors (6 BCC nearest neighbors and 8 BCC next-nearest neighbors) with very strict specifications of the distance to each of these neighbors. In liquid Si there are only four. Restrictions reduce the phase space available to the system and reduce its entropy. To ascertain an appropriate level of restriction imposed by the neighbors, we can be guided by the basic fact that each atom is specified by only three coordinates, often {x, y, z}. If the structural environment of an atom is described by coordinates shared with neighbors the number of shared coordinates needed to maintain the correct total number of coordinates, 3 N, is 6 shared coordinates at each atom. For example, crystal entropy is well represented by harmonic models based on a linear chain where the three components of the two vectors to neighbors along the chain comprise the six shared coordinates. In fluids, the RDF gives information only about scalar separations. For a fluid with a coordination of six, the distances to the six neighbors provides a good accounting of restricting coordinates; in such a liquid we expect the corrections to Kirkwood to be small. For fluids, e.g., Fe, with coordination greater than six too many constraints are imposed by Kirkwood and for coordination less than 6, such as Si, it is anticipated to under-restrict the structure. Here we propose a form of γ[g] that reflects our understanding of the trends with respect to coordination that need to be incorporated when the peaks of g(r) are narrow. For the differences in coordination number (C.N.) between different systems:

(28) $$\frac{1}{\gamma \left[g\right]}=1+\frac{c_{1}e^{-{\left(c_{2}\rho /\kappa \right)}^{2}}}{{\left(CN-6\right)}^{3}f_{p}}$$

The correction γ[g] was fit to the target entropy with the parameters $c_{1}$ and $c_{2}$ and then applied to the functional (5). As was the case for the parameters $q_{1}$ and $q_{2}$, $c_{1}$ and $c_{2}$ are optimized to the target entropy. It should be noted that while $q_{1}$, $q_{2}$, $c_{1}$, and $c_{2}$ all fit to the entropy obtained from thermodynamic integration, the three materials were fitted simultaneously with the goal of finding a universal set of these parameters that might serve a wide range of systems.

## 4. Results

### 4.1. Low and High Temperature Liquid Corrective Functionals

**Figure 3:**
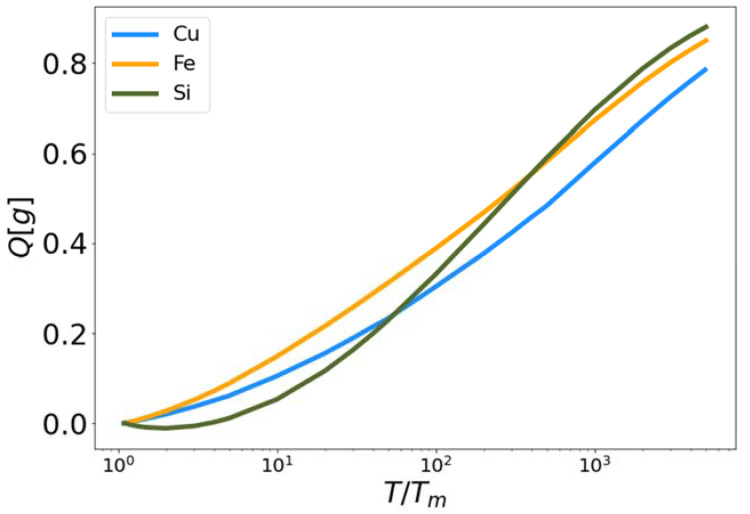
High temperature corrective functional Q vs. reduced temperature for MEAM copper and iron, and Tersoff silicon.

**Figure 4:**
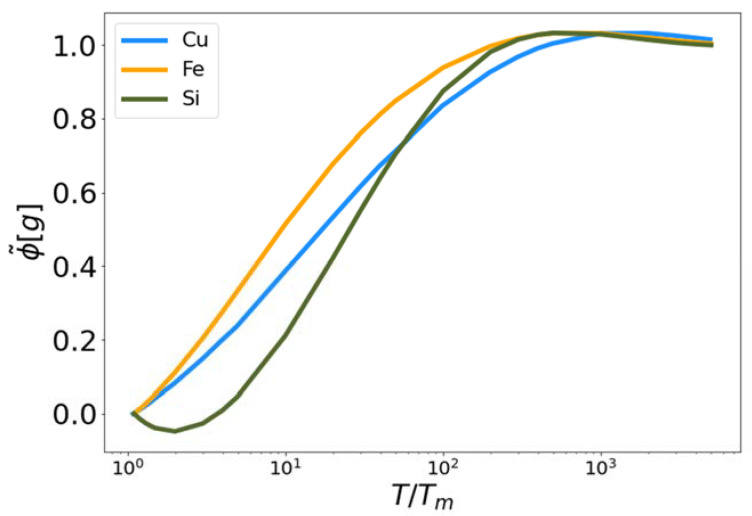
The fit high temperature corrective functional $\tilde{\phi }$ vs. reduced temperature for MEAM copper and iron, and Tersoff silicon.

**Figure 5:**
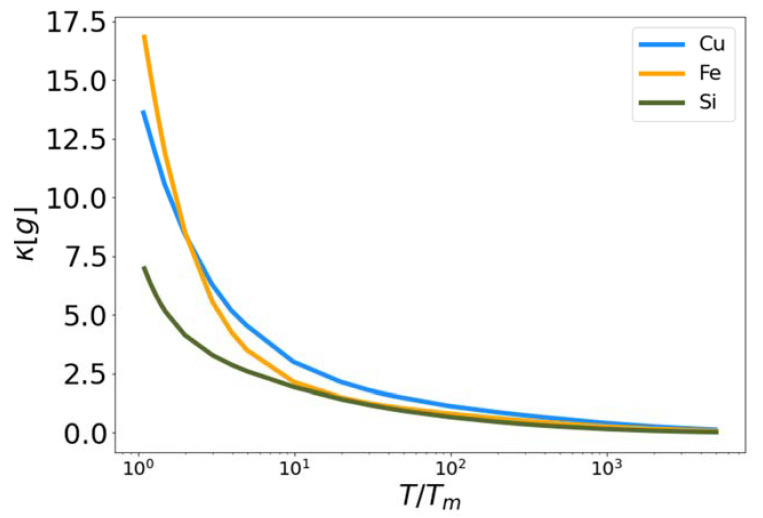
Low temperature corrective functional κ vs. reduced temperature for MEAM copper and iron, and Tersoff silicon.

The high and low temperature liquid correction functionals $\tilde{\phi }$ (Equation (23)) and γ (Equation (28)) were developed from their constituent functionals Q (Equation (21)) and κ (Equation (26)) respectively. The results are shown in Figure 2, Figure 3, Figure 4 and Figure 5. In Figure 3, the functional Q[g] is plotted for Cu, Fe and Si as a function of reduced temperature. In each case the functional approaches zero near the melting temperature (reduced temperature of unity) and approaches one as the temperature increases. The fact that Q[g] does not reach one, even at the highest temperatures simulated, indicates that 10^6^ K is not sufficient to force these materials (as governed by the MEAM and Tersoff interaction potentials) to behave as a perfect gas. Certainly Q[g] shares a similar qualitative shape for all three materials as a function of reduced temperature. To be clear, there are no fitting parameters in the functional Q[g].

In Figure 4, the functional $\tilde{\phi }\left[g\right]$ is plotted for Cu, Fe and Si as a function of reduced temperature. In each case the functional approaches zero near the melting temperature (reduced temperature of unity) and approaches one as the temperature increases. We observe that the functionals for Cu and Si exceed unity at intermediate temperatures. Again, the parameters, $q_{1}$ and $q_{2}$, were optimized to fit the excess entropy functional (Equation (5)) to the target entropy obtained via thermodynamic integration. The behavior of $\tilde{\phi }\left[g\right]$ is therefore a consequence of this optimization procedure.

The fitting constants $q_{1}$, $q_{2}$, $c_{1}$, and $c_{2}$ given in Table 2 are universal to the three systems examined here. It is evident from Table 2 that the EPFT provides a fivefold improvement over results based on Kirkwood entropy alone. For the two MEAM systems the maximum error in Kirkwood entropy is predicably at the PG limit where it approaches ~0.5 k_B_/atom. However, the maximum EPFT errors for these systems occur at intermediate temperatures. At the PG limit the error in the EPFT for the MEAM systems approaches 0 k_B_/atom.

In Figure 5, the functional κ[g] is plotted for Cu, Fe and Si as a function of reduced temperature. This functional characterizes pre-melting structure in the liquid at temperatures close to the melt temperature, so it should deviate from zero as the temperature decreases. In each case the functional approaches zero at high temperature and becomes positive as the temperature approaches the melt temperature (reduced temperature of unity). Again, this functional, with no fitting parameter, is qualitatively similar for all three materials, when plotted with respect to reduced temperature.

**Figure 6:**
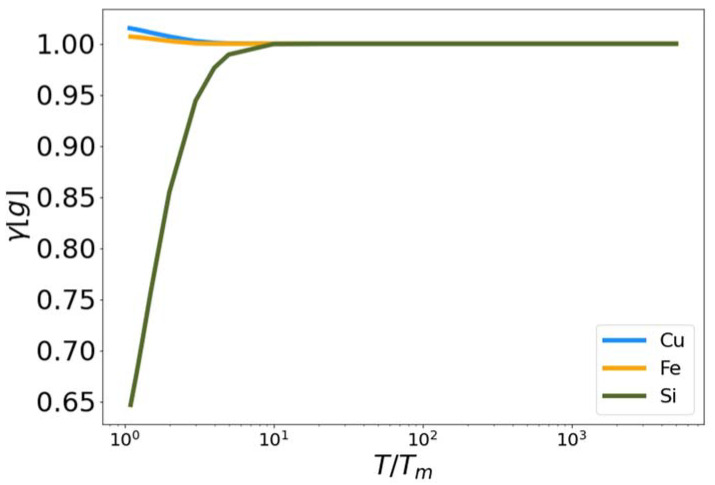
The fit low temperature corrective functional γ vs. reduced temperature for MEAM copper and iron, and Tersoff silicon.

In Figure 6, the functional γ[g] is plotted for Cu, Fe and Si as a function of reduced temperature. The purpose of this functional is to influence the calculated entropy near the melting temperature, so it should deviate from unity only where there is pre-melting structure in the liquid at temperatures close to the melt temperature. In each case the functional approaches unity at high temperature. However, the functional increases for Si while decreasing to differing extents for Fe and Cu. Again, the parameters $c_{1}$ and $c_{2}$ were optimized to fit the excess entropy functional (Equation (5)) to the target entropy obtained via thermodynamic integration.

The values of $c_{1}$ and $c_{2}$ are reported in Table 2. As with the parameters for the high temperature corrections, $c_{1}$ and $c_{2}$ are universal for the systems examined here.

### 4.2. Target Entropy

**Figure 7:**
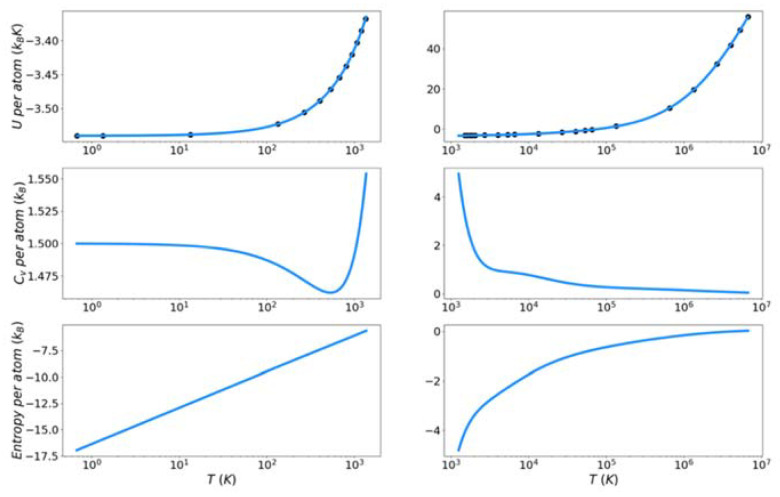
Thermodynamic integration development of solid and liquid entropy for copper.

**Figure 8:**
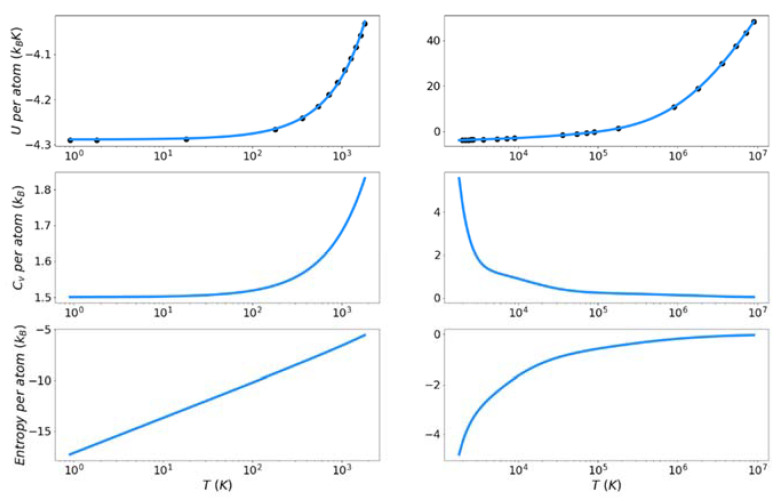
Thermodynamic integration development of solid and liquid entropy for iron.

**Figure 9:**
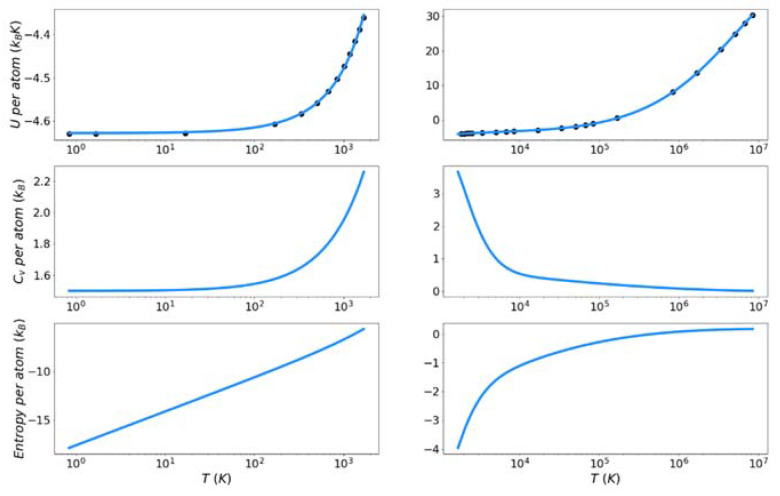
Thermodynamic integration development of solid and liquid entropy for silicon.

Figure 7, Figure 8 and Figure 9 show the solid and liquid target entropy developed as described in Section 3 for each of the three systems we have investigated. For each composite figure, the left column describes the solid and the right column the liquid. The x axis is reported in absolute temperature from 1 K to the melting temperature (solid) and from the melting temperature to nearly ten million K (liquid). Each column contains three figures, the potential energy (top), the excess constant volume heat capacity (middle) and the excess entropy (bottom).

Collectively we observe several features of these thermodynamic properties, which qualitatively validate the simulations. The potential energies for all materials monotonically increase with increasing temperature. The excess heat capacities are always positive and thus the excess entropy monotonically increases with increasing temperature. The excess heat capacities further demonstrate three qualities deemed to represent the physical system accurately. First, the solid heat capacity approaches 3/2 k_B_ on the approach to 0 Kelvin. Second, there is a sharp rise in heat capacity on the approach to the melt temperature in both the solid and liquid. Finally, the excess heat capacity approaches 0 as the temperature approaches infinity. These features provide confidence in the quality of the target entropies obtained through further thermodynamic integration.

The only anomalous behavior from these target thermodynamic properties is that we observe, for some cases, unexpected fluctuations in the slope of the heat capacity immediately before (Cu) and after the melting temperature (Cu, Fe). When the scale of the y-axis is taken into account, these fluctuations are deemed to be minor. They are artifacts of fitting discrete points of the potential energy to an integrable function.

In this work, the reference point for excess entropy is that it be zero at infinite temperature. To put the solid phase entropy on this same scale, the solid entropy must be shifted by a constant related to the entropy of fusion, as defined in Equation (13). It is important to remember that this constant is required only for the target entropy. It is not required by the entropy functional, which delivers an absolute excess entropy. These shift factors are reported in Table 3. The decomposition of the entropy shift into the three terms on the right-hand side of Equation (13) is also reported as fractions of the total.

### 4.3. Entropy Functionals

**Figure 10:**
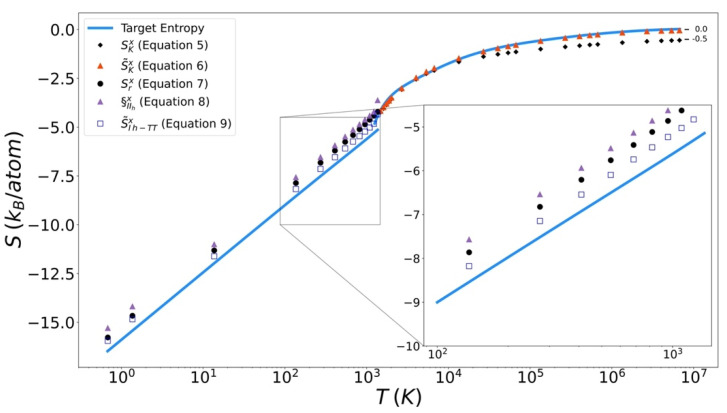
Thermodynamic integration development of solid Excess entropy of copper, comparing target entropy from thermodynamic integration with values from solid and liquid functionals.

**Figure 11:**
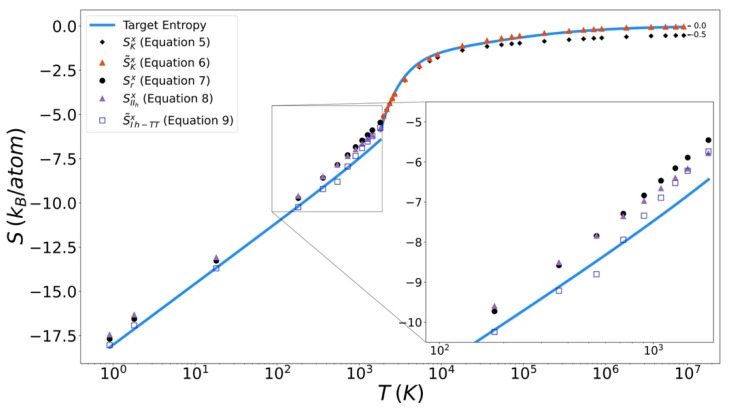
Thermodynamic integration development of solid Excess entropy of iron, comparing target entropy from thermodynamic integration with values from solid and liquid functionals.

Excess entropy from the functionals has been plotted with the target entropy. These results for copper, iron, and silicon are included in Figure 9, Figure 10 and Figure 11 respectively. Comparable plots for Johnson Fe appear in in Figure 1, Figure 2 and Figure 3 of Nicholson [14]. Collectively, in each plot, the reference entropy approaches zero in the high temperature limit. The excess entropy monotonically increases with increasing temperature. As a consequence of these two facts, the excess entropy is always negative.

On the liquid side, both the unmodified Kirkwood entropy (Equation (5)) and the modified Kirkwood entropy (Equation (6)), are plotted. In each case, the incorrect high temperature limit of the unmodified Kirkwood entropy is corrected by being shifted up a factor of ½ k_B_. At intermediate temperatures, the presence of the $\tilde{\phi }\left[g\right]$ in the modified Kirkwood formulation significantly improves the ability of the functional to describe the simulated entropy. Near the melt temperature, the presence of the κ[g], in the modified Kirkwood formulation, significantly improves the ability of the functional to describe the accelerated decrease in the simulated entropy.

**Figure 12:**
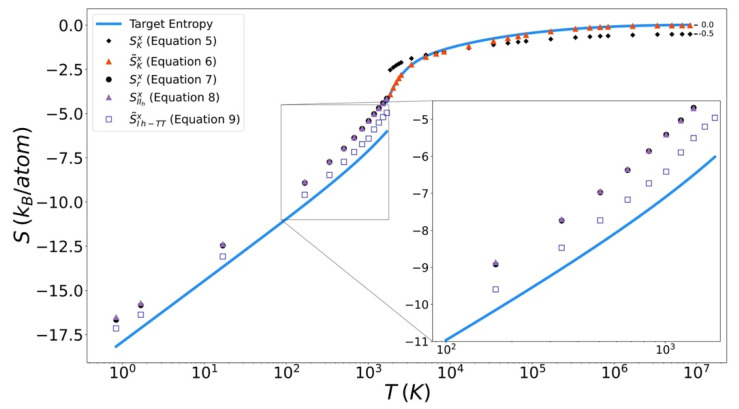
Thermodynamic integration development of solid Excess entropy of silicon, comparing target entropy from thermodynamic integration with values from solid and liquid functionals.

In Figure 12 it can be seen that in general the Kirkwood entropy for Si varies from the target entropy differently than is the case for the Kirkwood entropy for Cu and Fe. For temperatures approaching $T_{m}$ the Kirkwood entropy for Cu and Fe tend to undershoot the target while for Si it overshoots target entropy. There is also a subtle change in slope of the entropy for Si at intermediate temperatures in modified Kirkwood entropy, that is not evident in the Cu and Fe entropies.

On the solid side of the curve, we compare the target entropy with the three versions of the solid entropy functional explored in this work (Equations (7)–(9)). All models produce quantitatively similar results to the target entropy obtained from thermodynamic integration. The slope is well captured. The degree to which the intercept is captured varies. For Cu (Figure 10), Fe (Figure 11), and Si (Figure 12), Equation (9) gives the best fit. It is further observed that the trend in relative entropies from Equations (7)–(9) is not the same for all three materials.

## 5. Discussion

Table 2 shows that EPFT provides a significant reduction in error over the unmodified Kirkwood entropy. The error for the entropy of the EPFT compared to thermodynamic integration is reported in Table 2 and is below 0.1 k_B_/atom for all three systems. In the case of silicon this represents an order of magnitude improvement over Kirkwood entropy.

The lower performance of Kirkwood entropy for the Tersoff silicon system most likely originates from the construction of the Tersoff potential, which takes into account multibody interactions through angle-dependent terms. As discussed, the Kirkwood entropy is ultimately a sub-functional of the EPFT and assumes pair potentials only. This, in fact, is the reason the multibody Tersoff potential was chosen, to test the compatibility of functionals accounting for only pair correlations with a multibody interaction potential. The new forms of $\tilde{\phi }\left[g\right]\;$and γ[g] presented take into account the effects of coordination number and density. These forms bring us closer to an accurate pair entropy functional and, therefore, overcome some of the limitations of Kirkwood entropy when applied to multibody potential systems.

It is well known that entropy should approach zero as temperature approaches absolute zero. The reader is reminded of two important restrictions we have imposed on this work. First, we are only addressing excess configurational entropy. We have developed the target entropy for this purpose from only the potential energy of the systems. This can be seen in the fact that the solid heat capacity approaches $3/2\;k_{B}$ as temperature approaches 0 Kelvin. By way of the equipartition theorem, this represents half of the $3\;k_{B}$ dictated by the law of Dulong Petit. Second, this work is based on classical MD simulations that do not take into account any quantum effects that begin to dominate as temperature approaches 0 K. A similar examination of the high temperature limit can be used to confirm the expected result of excess entropy approaching zero as the system approaches a configuration equivalent to that of a perfect gas.

## 6. Conclusions

The goal of this work was to explore the universality of a recently published Entropy Pair Functional Theory (EPFT) and its applicability to many-body interactions. The EPFT demonstrated that it could accurately describe materials obeying classical many-body (non-pairwise) potentials (MEAM and Tersoff) and we can expect that it would apply equally well to evaluation of the entropy to other many-body potentials, including first principles simulations. The practical value of EPFT is its potential to deliver excess entropy from a single simulation. A suite of classical molecular dynamics simulations was performed for Cu (FCC, MEAM), Fe (BCC, MEAM) and Si (diamond cubic, Tersoff) over a temperature range from 1 K to over 10^6^ K. Using thermodynamic integration, the excess entropy was calculated across this temperature range. The universality of EPFT was investigated by comparing the excess entropies of these materials from EPFT with the standard obtained from thermodynamic integration over the entire temperature range. The EPFT approach provides a significant improvement over Kirkwood entropy, yielding average errors of 0.06, 0.08, and 0.05 k_B_/atom for Cu, Fe and Si, respectively.

As presented here, the EPFT approach to computing liquid phase excess entropies contains four parameters that are fit to simulation data obtained across a temperature range. These parameters are universal and can be made more robust as simulation data from additional systems is added to the data pool. Of note, the EPFT formalism for the solid phase contains no adjustable parameters and is universal to within the accuracy reported above. Utilization of EPFT to explore entropies of defective crystals and high entropy alloys is underway.

## Figures and Tables

**Table 1:** Molecular Dynamics Simulations Parameters.

System	T_melt_ (K)	Structure	Potential	N(Atoms)	Box Size (Å^3^)	𝜌 (N/Å^3^)
Cu	1347	FCC	MEAM	87,808	1,041,357.395	8.432 × 10^−2^
Fe	1812	BCC	MEAM	93,312	1,081,182.881	8.631 × 10^−2^
Si	1687	Diamond Cubic	Tersoff	54,872	1,100,297.642	4.987 × 10^−2^

**Table 2:** Summary of fit parameters and errors of EPFT and Kirkwood entropy functionals. Error is reported as the difference between the target entropy and the functional entropy. The fit parameters are unitless.

System	*q*_1_	*q*_2_	*c*_1_	*c*_2_	Kirk. Avg. Err (k_B_/Atom)	EPFT Avg. Err (k_B_/Atom)
Cu					0.32	0.06
Fe	3.24834	−2.406550	−320.1305	1.02966	0.41	0.08
Si					0.61	0.05

**Table 3:** Excess entropy shift between solid and liquid phases and its decomposition.

System	$\Delta S_{tot}$ (J/mol/K)	$\Delta S_{L}$ (Fraction)	$\Delta S_{s}$ (Fraction)	$\Delta S_{L\to S}$ (Fraction)
Cu	−7.03	−0.930	0.540	1.390
Fe	−4.27	−1.177	0.743	1.434
Si	−13.82	−0.016	−0.009	1.025

## Data Availability

Data is available upon request from D.J.K.

## Appendix A

Equation of the form (A1) was used to fit potential energy where the order *i* is increased as necessary to obtain properly behaved heat capacity functions but not to exceed the number of potential energy data points.

(A1) $$U=\underset{i}{\sum }a_{i}ln{\left(T\right)}^{i}$$

The constant volume heat capacity was determined as follows.

(A2) $$C_{v}\equiv {\left(\frac{\partial U}{\partial T}\right)}_{v}$$

(A3) $$C_{v}=\underset{i}{\sum }\frac{ia_{i}ln{\left(T\right)}^{i-1}}{T}$$

The target excess entropy, by which the EPFT was validated, was determined as follows.

(A4) $$S\equiv \int_{T_{ref}}^{T}\frac{C_{v}}{T^{\prime }}dT\prime$$

(A5) $$S=\underset{i}{\sum }\int_{T_{ref}}^{T}\frac{ia_{i}ln{\left(T^{\prime }\right)}^{i-1}}{T^{\prime 2}}dT^{\prime }=\underset{i}{\sum }ia_{i}\int_{T_{ref}}^{T}\frac{ln{\left(T^{\prime }\right)}^{i-1}}{T^{\prime 2}}dT^{\prime }$$

## Appendix B

To determine the likelihood that a trial volume contains only one atom we determine the probability that N−1 atoms in the system are outside of the trial volume. For a system of *N* atoms and trial volume $V=4/3\pi r^{3}$ and a total system volume $V_{s}=4/3\pi R_{s}^{3}$ the probability that any one of the *N* atoms is outside the trial volume is

(A6) $$p^{o1}\left(r\right)=1-\frac{4/3\pi r^{3}}{N4/3\pi R_{s}^{3}}\;$$

The joint probability that all but one of the atoms in the system are outside the trial volume then becomes

(A7) $$p^{o}\left(r\right)={\left(1-\frac{r^{3}}{NR_{s}^{3}}\right)}^{N-1}$$

The binomial theorem can be used to expand equations of this form. Setting $a=r^{3}/R_{s}^{3}$

(A8) $$p^{o}\left(r\right)=\underset{N\to \infty }{\lim}{\left(1-\frac{a}{N}\right)}^{N-1}=1-\frac{a}{1!}+\frac{a}{2!}-\frac{a}{3!}\;$$

This series is recognizable as the expansion of $e^{a}$ and forms the basis for the high temperature correction Q[r].

(A9) po(r)=exp(−a)=exp(−r3Rs3) 
